# Exceptionally prolonged retention of a silicone-covered esophageal stent before TIPS for refractory variceal bleeding: a case report

**DOI:** 10.3389/fmed.2026.1927571

**Published:** 2026-09-07

**Authors:** Xiulan Peng, YuanYuan Wang, Juping Han, PingXiao Huang

**Affiliations:** 1 Department of Oncology, Geriatric Hospital Affiliated to Wuhan University of Science and Technology, Wuhan, Hubei, China; 2 Department of Gastroenterology, The Second Affiliated Hospital of Jianghan University, Wuhan, Hubei, China

**Keywords:** prolonged indwelling, refractory esophageal variceal bleeding, self-expanding metal stents, surgery, transjugular intrahepatic portosystemic shunt

## Abstract

**Background and aims:**

Refractory esophageal variceal bleeding (REVB) in patients with cirrhosis is associated with high mortality. Self-expanding metal stents (SEMS) serve as a salvage therapeutic option. However, data regarding their prolonged use as a bridge to transjugular intrahepatic portosystemic shunt (TIPS) remain limited. We report a case in which REVB was managed with prolonged SEMS placement before TIPS.

**Case presentation:**

A 72-year-old female with decompensated cirrhosis presented with acute hematemesis. After failure of endoscopic injection sclerotherapy and band ligation to control active bleeding from a ruptured esophageal varix, a fully covered SEMS was successfully deployed. The patient declined the scheduled stent removal and was subsequently followed conservatively. TIPS was performed 387 days after stent placement, followed by a successful stent retrieval. Clinical outcomes, including rebleeding episodes and procedure-related complications, were recorded throughout follow-up until July 26, 2026.

**Results:**

Immediate hemostasis was achieved following SEMS deployment. During the 387-day indwelling period, the patient developed intermittent acid regurgitation, retrosternal discomfort, and endoscopic evidence of mucosal ulceration and granulation tissue at the stent site. The stent was successfully removed after TIPS, and follow-up endoscopy revealed complete resolution of the esophageal varices. The patient remained free of recurrent bleeding till the last follow-up. However, two episodes of hepatic encephalopathy occurred after TIPS.

**Conclusion:**

This case suggests that successful removal of a fully covered esophageal SEMS may remain technically feasible even after an exceptionally prolonged retention period. Nonetheless, this observation should not be interpreted as support for routine prolonged stent placement, which lies outside current guideline recommendations and may entail substantial risks.

## Introduction

Esophagogastric varices develop in 30–40% of patients with compensated cirrhosis and in more than 60% of those with decompensated disease (1). In the 1980s and 1990s, six-week mortality following acute variceal bleeding was reported to be as high as 40–60% (2). Despite substantial advances in pharmacological and endoscopic therapies in recent decades, mortality remains considerable with contemporary rates estimated at 15–20% (3). Furthermore, among patients without secondary prophylaxis, the six-week risk of rebleeding after initial bleeding is 15–20%, thus increasing to 60% within the first year (4). Although the incidence of viral hepatitis-related cirrhosis has declined, the increasing prevalence of non-alcoholic fatty liver disease and alcohol-related cirrhosis in many regions suggests that the global burden of cirrhosis—and consequently the population at risk for variceal bleeding—will continue to grow over the next two decades (5–8).

Current management of esophagogastric variceal bleeding (EGVB) can be divided into two phases, viz., control of acute bleeding and prevention of recurrence. Therapeutic options include volume resuscitation, pharmacotherapy, endoscopic interventions, radiological procedures, and surgery (9–12). Several modalities for acute esophageal variceal bleeding have been described, including endoscopic band ligation, injection sclerotherapy, Sengstaken–Blakemore tube placement, self-expanding metal stents (SEMS), and transjugular intrahepatic portosystemic shunt (TIPS) (13). In this report, we describe a case of refractory esophageal variceal bleeding (REVB) that was successfully managed with exceptionally prolonged use of a silicone-covered SEMS during the acute bleeding phase, followed by the subsequent TIPS.

## Case report

A 72-year-old female with a history of decompensated cirrhosis for more than 8 years presented to our hospital with acute upper gastrointestinal bleeding. She had been hospitalized on five prior occasions for active esophageal variceal bleeding and had undergone endoscopic injection sclerotherapy with tissue glue for esophageal varices 3 months earlier. On October 31, 2024, approximately 1 h post dinner, the patient developed persistent dull pain and discomfort in the upper abdomen, followed by a single episode of vomiting, approximately 150 mL of bright red blood. Physical examination revealed marked tenderness in the upper abdomen. The relevant clinical and laboratory findings are summarized in Tables 1, 2. The initial diagnosis was observed as active esophageal variceal bleeding.

**Table 1:** Blood routine test results that obtained at initial admission.

Lab measure	Values	Normal reference value
Erythrocytes	2.88 × 10^12^/L	3.5–5.5 × 10^12^/L
Hemoglobin	58 g/L	115–150 g/L
Hematocrit	20.30%	37–47%
Leukocytes	2.63 × 10^9^/L	3.5–9.5 × 10^9^/L
Thrombocytes	52 × 10^9^/L	125–350 × 10^9^/L

**Table 2:** Child-Pugh scoring system of pre-SEMS and pre-TIPS.

Clinical/lab measure	Pre-SEMS		Pre-TIPS	
	Measure	Points	Measure	Points
Encephalopathy	None	1	None	1
Ascites	Moderate	2	Severe	3
Bilirubin	< 34	1	< 34	1
Albumin	30.6	2	35.2	1
Prothrombin time (PT)/ (INR or seconds prolonged)	17.3/1.38	1	16.7/1.31	1
Total		7		7
Child-Pugh class		B		B

The patient was admitted to the intensive care unit and received vital sign monitoring, vasopressor support, fluid resuscitation, acid suppression (proton pump inhibitor), portal pressure reduction (octreotide), antibiotic therapy (ceftriaxone sodium), and blood product transfusions (packed red blood cells, plasma, and cryoprecipitate). Despite pharmacological management, the patient experienced recurrent bleeding.

**Figure 1:**
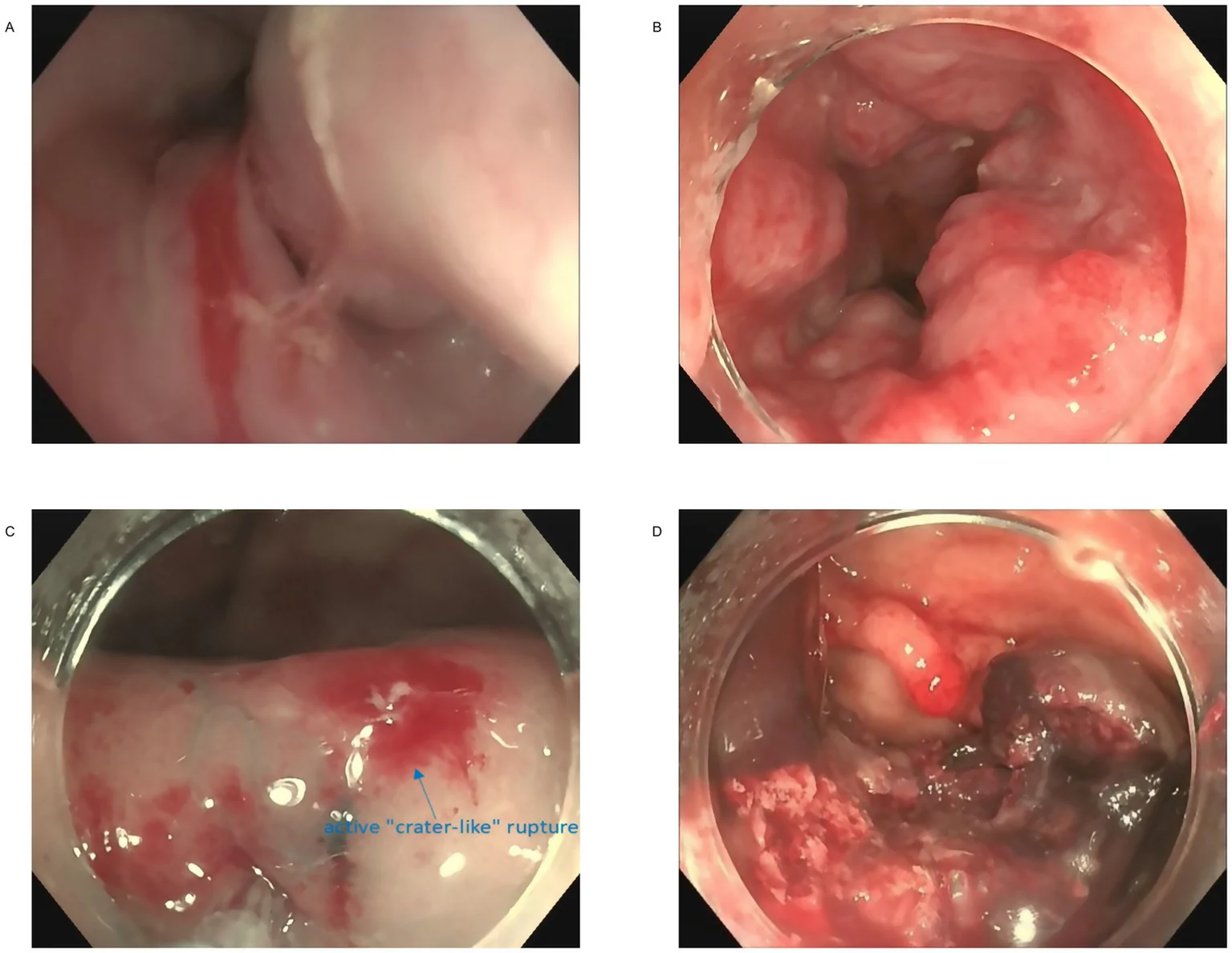
Endoscopic images showing the diagnosis of REVB. **(A)** Active bleeding from the esophagus; **(B)** Esophageal varices visualized following flush irrigation; **(C)** The variceal veins and bleeding varix, **(D)** Large amounts of blood clots were observed in the gastric cavity.

**Figure 2:**
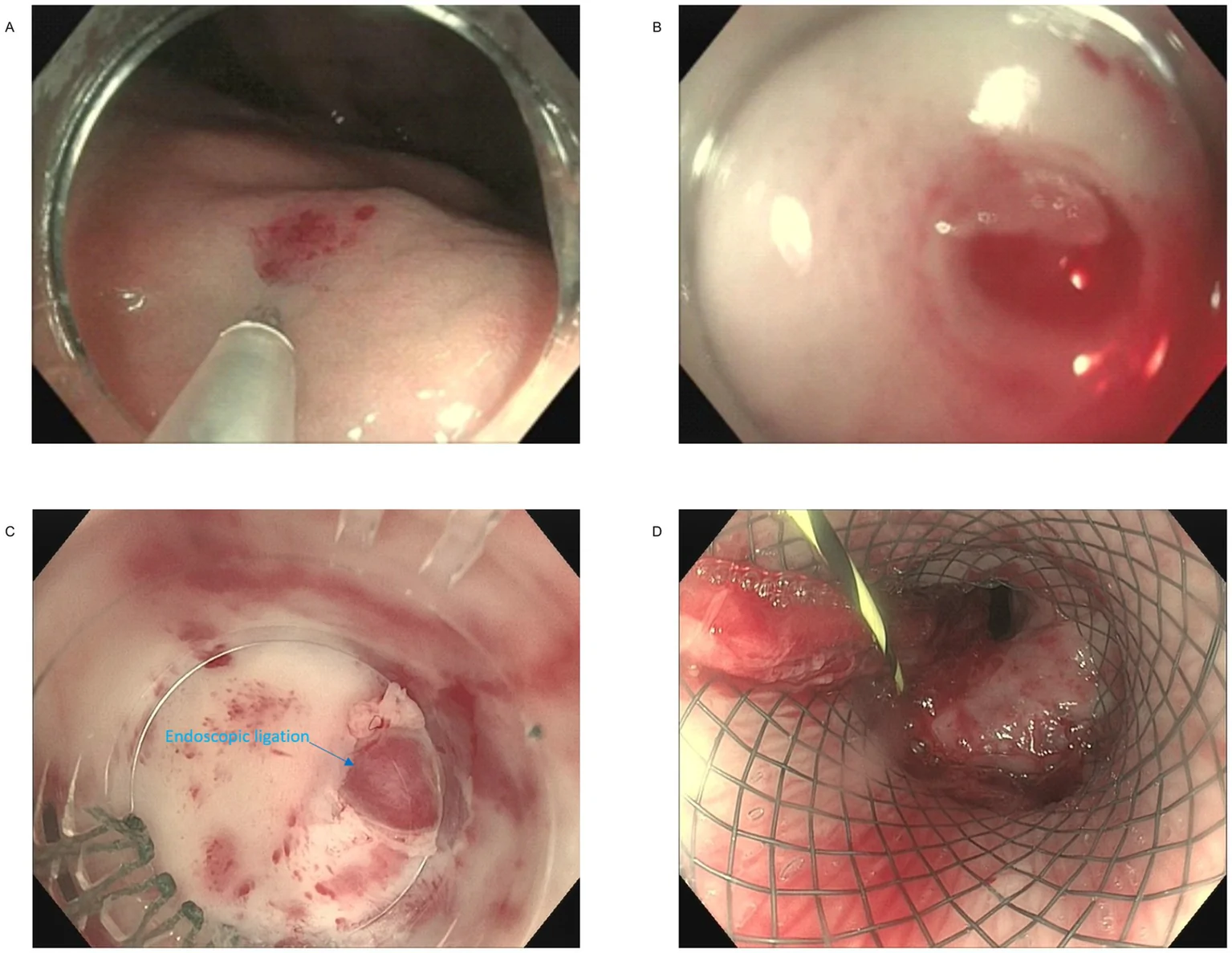
Endoscopic images showing treatment of REVB. **(A)** Injection of sclerosant around the bleeding point under direct endoscopic visualization. **(B)** Active oozing hemorrhage originating from the esophagus. **(C)** Endoscopic variceal band ligation performed to achieve hemostasis. **(D)** Endoscopic view after SEMS implantation.

On November 1, 2024, under general anesthesia with endotracheal intubation, an endoscope was advanced to the descending duodenum. Large amounts of blood clots and fresh blood adhering to the gastric cavity were observed. Multiple tortuous, serpentine varices were visible from 25 cm below the incisors in the esophagus, extending to the cardia (40 cm from the incisors), with the largest diameter measuring approximately 3 cm. Several varices exhibited red-color signs (Figures 1A,B). At the lower esophagus near the cardia, an active “crater-like” rupture was observed, accompanied by active spurting and oozing of blood (Figure 1C). Multiple active bleeding sites were also noted in the upper gastric body (Figure 1D). A transparent cap was attached, and endoscopic injection of tissue adhesive and sclerosant was performed in the esophagus and gastric body using a disposable Boston Scientific injection needle, polidocanol, and Kangpaite tissue adhesive (Figure 2A). Following this treatment, bleeding from the gastric body lesions ceased. However, active hemorrhage persisted at the rupture site in the lower esophagus (Figure 2B). Endoscopic ligation was subsequently performed but failed to achieve adequate hemostasis (Figure 2C). After the consent was obtained from the patient’s family during the procedure—and given that the TIPS was declined due to financial constraints—a fully covered self-expanding metal stent [SEMS; silicone-covered esophageal stent system, REF: MTN-SE-S-18/60-A-8/650, Micro-Tech (Nanjing) Co., Ltd.] was implanted under direct endoscopic visualization for compression hemostasis. The procedure was uneventful, and bleeding ceased following SEMS deployment (Figure 2D; Supplementary Video S1). A nasogastric tube was then placed under direct visualization at 20 cm from the incisors. Post-operatively, the patient received hemostatic therapy, fluid resuscitation, blood transfusion, and nutritional support. The bleeding gradually resolved, and the clinical symptoms improved markedly. The patient was successfully discharged on the 25th day post-procedure.

**Figure 3:**
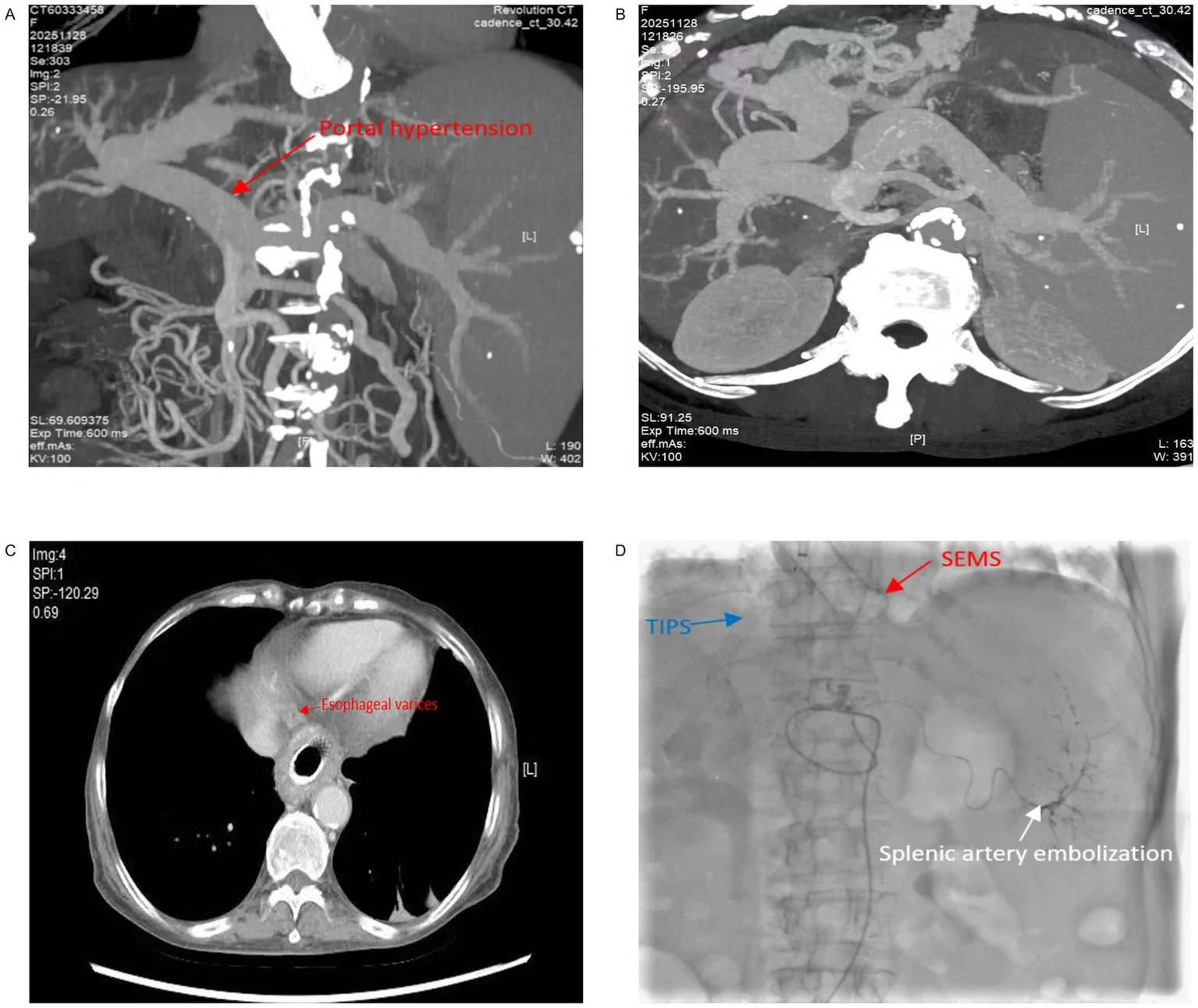
Contrast-enhanced CT scans findings before the TIPS procedure. **(A)** Pre-TIPS portal vein CT venography (CTV) review; **(B)** The portal vein and its branches; **(C)** Esophageal varices; **(D)** Post-TIPS imaging.

It was recommended that the SEMS be removed after TIPS during follow-up; however, the patient refused the procedure (Figure 3). Following SEMS placement, she experienced intermittent acid regurgitation and retrosternal discomfort, which were relieved with oral vonoprazan and hydrotalcite. During this period, no further episodes of gastrointestinal bleeding occurred. On November 23, 2025, follow-up portal vein CT venography (CTV) revealed dilatation of the portal vein and its intrahepatic branches, esophageal and gastric varices, and recanalization of the umbilical vein. Compared with prior imaging, a tubular SEMS shadow was observed in the lower esophagus, with increased haziness of the surrounding fat planes (Figures 3A–C). Pre-TIPS clinical and laboratory data are presented in Tables 2, 3.

**Table 3:** Clinical/lab measure of pre-SEMS and pre-TIPS.

Clinical/lab measure	Pre-SEMS	Pre-TIPS	Normal reference value
Bilirubin	12.8	31.4	0–21umol/L
INR	1.38	1.31	0.8–1.2
Albumin	30.6	35.2	35–55 g/L
Creatinine	56	73	41–97umol/L
Sodium	140.4	139.3	135–145 mmol/L
Ammonia	59	60	18–72umol/L
Blood urea nitrogen	5.9	6.0	1.7–8.3 mmol/L
MELD/MELD-Na score	8/7	7/6	–

**Figure 4:**
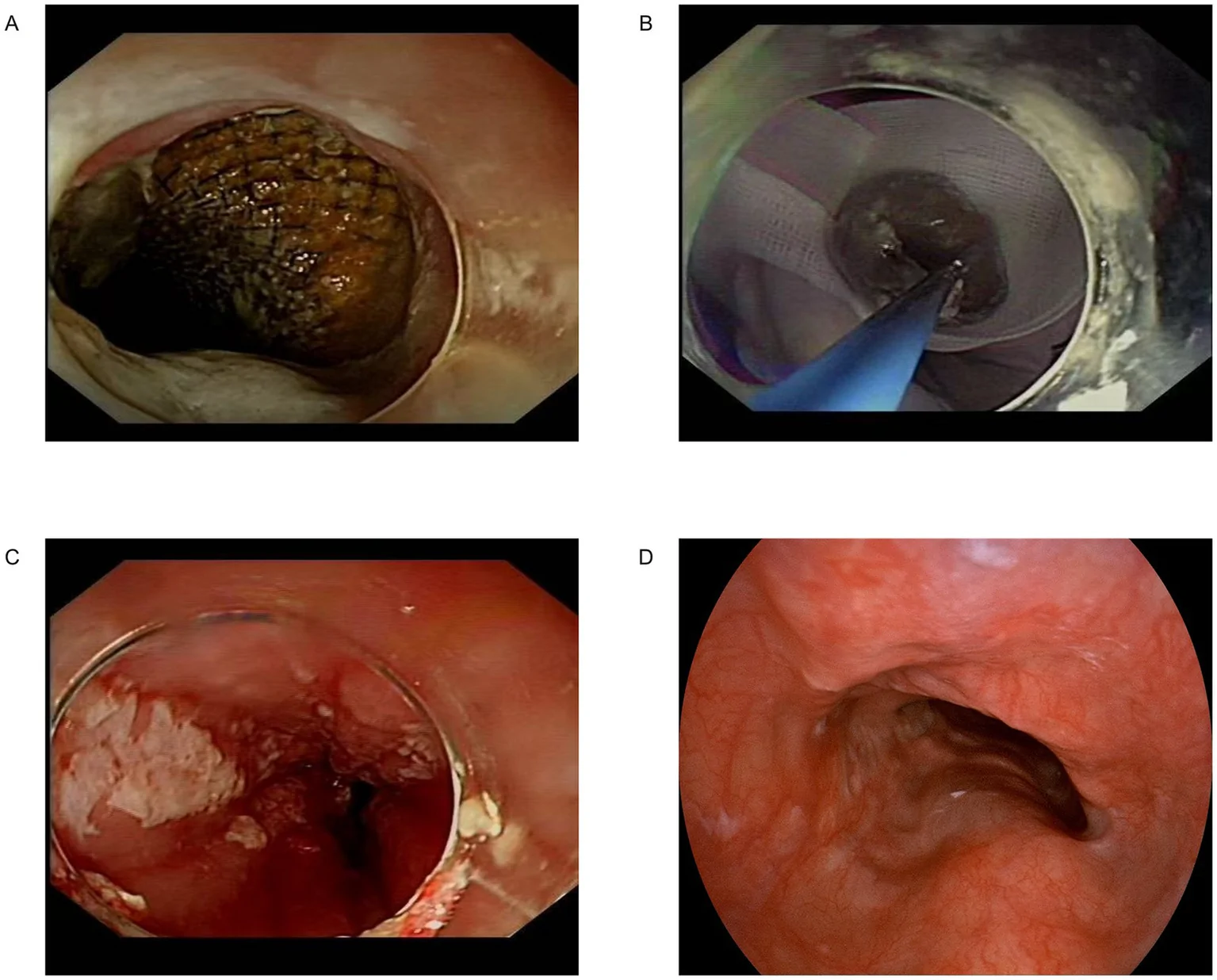
Endoscopic findings before and after stent removal and at 1 week post-removal. **(A)** Before stent removal; **(B)** Endoscopic removal of the stent; **(C)** After stent removal; **(D)** Endoscopic re-examination at 1 week post-TIPS showing disappearance of the original variceal veins.

On November 30, 2025, because the costs of TIPS became more affordable, her family opted for TIPS (Model PTB8107275W, W. L. Gore & Associates, Inc.) (Figure 3D), gastric coronary vein embolization, splenic artery embolization, and endoscopic SEMS removal (Figures 4A,B). Inspection of the SEMS lumen revealed no impacted food debris, necrotic tissue, or secretions; however, mucosal ulceration and granulation tissue were noted on the esophageal wall (Figure 4C). One week later, follow-up endoscopy showed no evidence of bleeding, and the previously noted varices had resolved (Figure 4D; Supplementary Video S2). No recurrent gastrointestinal bleeding occurred following the TIPS procedure. The patient developed two episodes of hepatic encephalopathy after TIPS (December 2025 and May 2026), both of which were successfully managed with inpatient treatment. She remained under continuous follow-up surveillance as of July 26, 2026.

## Discussion

Patients with EGVB who undergo emergency endoscopy as early as possible identify the bleeding source and facilitate timely therapeutic intervention (9, 10). Endoscopic modalities, including endoscopic variceal ligation (EVL) and endoscopic injection sclerotherapy (EIS), represent the first-line treatment for EGVB (14). However, acute variceal bleeding fails to be controlled within 6 h after the combination of pharmacologic and endoscopic therapy in 10–20% of cases, which represents a condition defined as REVB and constitutes a medical emergency requiring prompt salvage measures (15). Therapeutic options for REVB include balloon tamponade with a Sengstaken–Blakemore tube, TIPS, SEMS, and surgical intervention (16). We report a case in which a fully covered SEMS was successfully used with exceptionally prolonged retention prior to TIPS for REVB, based on the patient’s treatment preference.

Although balloon tamponade achieves hemostasis in up to 80% of acute variceal bleeding episodes, it has significant limitations. It requires intensive care monitoring, cannot remain in place beyond 24 h, and is associated with a high risk of rebleeding after balloon deflation (17, 18). Furthermore, balloon tamponade is linked to high rates of adverse events, including esophageal necrosis, perforation, and aspiration pneumonia, with an associated mortality rate approaching 20% (12, 19). In recent years, fully covered SEMS have emerged as a novel alternative (20). Originally developed for malignant dysphagia, SEMS has increasingly been recognized as more effective and a safer option. SEMS acts as a bridge to TIPS or liver transplantation, offering advantages over balloon tamponade, including the ability to remain *in situ* for longer periods and to permit oral nutrition. Results from a meta-analyses incorporating 5 studies with a total of 80 patients indicated that treatment with self-expanding metal stents (SEMS) for acute esophageal variceal bleeding achieved a success rate of stent deployment was 96.7% and complete response to esophageal stenting was in 93.9%. The incidence of rebleeding was 13.2% and the overall mortality was 34.5% (21). A 2024 systematic review and a meta-analysis encompassing 12 studies with 225 patients reported a pooled immediate bleeding control rate of 91% for SEMS in REVB, with rebleeding, stent ulceration, and migration rates of 17, 7, and 18%, respectively (22). Moreover, SEMS placement for REVB is clinically feasible and achieves high success rates (21–23). Our case is consistent with these reports, demonstrating that SEMS effectively controlled REVB and provided a bridge to subsequent TIPS.

For the treatment of gastroesophageal variceal bleeding with fully covered SEMS, the currently recommended retrieval time in clinical practice is generally 7–14 days after placement, with an optimal target of not more than 7 days (24); however, in clinical studies, actual indwelling times vary. A 2024 multicenter study reported a median SEMS retrieval time of 8 days (25), another reported an indwelling time of up to 214 days in a patient who was unsuitable for TIPS or liver transplantation (26). In rare cases where TIPS is not feasible—for example, owing to portal vein tumor thrombus—stents have been reported to be left in place long term as a final palliative measure (27). In our case, the SEMS was successfully removed 387 days after placement and subsequent to TIPS. To our knowledge, this represents the longest case identified in the published literature. A 2024 systematic review on long-term esophageal stent use across multiple indications revealed that extended dwelling times did not significantly increase complication rates, suggesting that stents may be used safely as a longer-term bridge (28). While effective and generally safe, SEMS placement is not without limitations. Stent migration can occur, necessitating vigilant monitoring and potential repositioning (29). Esophageal ulceration or mucosal trauma may develop, particularly with prolonged indwelling times, underscoring the importance of careful monitoring (22, 27). In the present case, after SEMS implantation, the patient experienced intermittent acid regurgitation and retrosternal discomfort, which were relieved with oral vonoprazan and hydrotalcite. Follow-up CT revealed increased fat stranding around the esophagus, which may indicate an inflammatory reaction following SEMS placement. After SEMS extraction, mucosal ulceration and granulation tissue were observed on the esophageal wall. Nevertheless, no major stent-related complications requiring intervention occurred during the indwelling period.

TIPS reduces portal pressure by creating a shunt that connects the high-pressure portal vein to the lower-pressure hepatic vein, thereby bypassing hepatic resistance. As a salvage therapy for patients for whom medical and/or endoscopic treatment has failed, TIPS has been shown to achieve effective hemostasis in more than 90% of patients (30). Initially used for the management of REVB as a salvage or rescue procedure, TIPS has since been employed for the prevention of rebleeding and as secondary prophylaxis (31). The 2025 EASL Clinical Practice Guidelines on TIPS provide updated recommendations on patient selection, emphasizing that the MELD score remains the most accurate predictor of post-TIPS survival and that a multidisciplinary approach—rather than an absolute MELD cutoff— is recommended for TIPS candidacy assessment (32). However, because toxic enteric substances that would normally be cleared by hepatic metabolism are shunted directly into the systemic circulation, the major complication of TIPS is hepatic encephalopathy. Other less common complications include shunt dysfunction because of the occlusion, cardiac overload, new-onset or worsening pulmonary hypertension, and liver failure (33). In our patient, TIPS was uneventfully performed, after which the SEMS was successfully removed. Endoscopic disappearance of varices was observed 1 week after TIPS, which may reflect decompression or collapse rather than permanent anatomical resolution. No further gastrointestinal bleeding occurred after the procedure; however, the patient experienced two episodes of hepatic encephalopathy post-procedurally, both of which were successfully managed with inpatient treatment.

Both SEMS and TIPS can serve as effective hemostatic measures for REVB; however, the optimal timing and patient selection for esophageal stenting versus early TIPS remain debated. An open-label randomized controlled trial (TIPSEMS-VB trial) directly compared TIPS (*n* = 20) with SEMS (*n* = 21) in patients with REVB or post-endoscopic variceal band ligation (EVL) ulcer bleeding that was not controlled by endoscopic therapy or rebled after treatment. Six-week mortality was 52.4% in the SEMS group versus 10% in the TIPS group (*p* = 0.015), with significantly higher and early rebleeding rates in the SEMS arm (23.8% vs. 5%). The incidence of new-onset sepsis was higher in the SEMS arm (47.6% vs. 15%), although the incidence of hepatic encephalopathy (≥grade 2) (9.5% vs. 20%) and ischemic hepatitis (4.76% vs. 20%) was lower in the SEMS group (34). The Baveno VII consensus recommends TIPS as the preferred rescue therapy for REVB after combined pharmacologic and endoscopic therapy, with SEMS serving as a temporary bridge therapy allowing time for TIPS or liver transplantation (9). The updated 2025 APASL guidelines on acute variceal bleeding recommend SEMS as a salvage modality when TIPS is not immediately available but emphasize that TIPS remains the preferred definitive rescue therapy (13). These guidelines collectively underscore TIPS as a superior salvage therapy when feasible. The final decision should be individualized based on the patient’s clinical condition, as well as the available institutional resources and operator expertise.

In our case, TIPS was indicated for this patient; however, the procedure initially declined, and consent was obtained for SEMS implantation instead. It was recommended that the esophageal stent be removed within 1 month, but the patient again refused the procedure. Consequently, the SEMS remained *in situ* for 387 days prior to TIPS. Several factors may have collectively mitigated the risk of adverse events. First, the use of a fully covered silicone SEMS, combined with the absence of SEMS migration, likely reduced the risk of mechanical injury and obstruction or rebleeding. Second, the relatively preserved liver function suggested adequate hepatic compensatory capacity. Third, continuous acid suppression may have alleviated local inflammation, while close clinical follow-up enabled timely detection and management of potential complications. Nevertheless, it should be emphasized that these are only possible explanations and should not be construed as evidence supporting routine prolonged SEMS placement. Further studies with larger sample sizes are warranted to validate these observations.

Given the limited sample size of long-term indwelling studies in the current SEMS literature and the lack of long-term data regarding rebleeding rates and safety, TIPS remain the first-line salvage treatment and an effective form of secondary prophylaxis. For REVB patients with a limited life expectancy or poor hepatic function that confers a high risk of hepatic encephalopathy, the potential use of SEMS should currently be restricted to highly selected cases or clinical trial settings and cannot be recommended as a general alternative. Additional randomized controlled trials are needed to establish precision treatment strategies for REVB and to further define the comparative efficacy of SEMS and TIPS in specific patient subgroups.

## Conclusion

This case suggests that successful removal of a fully covered esophageal SEMS may remain technically possible even after exceptionally prolonged retention. However, it should not be interpreted as evidence supporting routine prolonged stent placement, which remains outside current guideline recommendations and may carry substantial risks.

## Data Availability

The raw data supporting the conclusions of this article will be made available by the authors, without undue reservation.

## Supplementary material

SUPPLEMENTARY VIDEO S1 Demonstrates the self-expanding covered silicone-coated esophageal metal stent after implantation.

SUPPLEMENTARY VIDEO S2 Endoscopic re-examination 1 week after TIPS: regression of the original varicose veins.

SUPPLEMENTARY FIGURE S1 Clinical course timeline of the patient.

SUPPLEMENTARY FIGURE S2 **(A,B)** Endoscopic images at 3 months after SEMS implantation.
